# Biomarkers for Temporomandibular Disorders: Current Status and Future Directions

**DOI:** 10.3390/diagnostics10050303

**Published:** 2020-05-15

**Authors:** Abdalwhab Zwiri, Mohammad A. I. Al-Hatamleh, Wan Muhamad Amir W. Ahmad, Jawaad Ahmed Asif, Suan Phaik Khoo, Adam Husein, Zuryati Ab-Ghani, Nur Karyatee Kassim

**Affiliations:** 1 School of Dental Sciences, Health Campus, Universiti Sains Malaysia, Kubang Kerian 16150, Kelantan, Malaysia; dr.zwiri@gmail.com (A.Z.); wmamir@usm.my (W.M.A.W.A.); jawaad@usm.my (J.A.A.); adamkck@usm.my (A.H.); 2 Department of Immunology, School of Medical Sciences, Health Campus, Universiti Sains Malaysia, Kubang Kerian 16150, Kelantan, Malaysia; m7md.7tml@gmail.com; 3 Hospital Universiti Sains Malaysia, Kubang Kerian 16150, Kelantan, Malaysia; 4 Department of Oral Diagnostic and Surgical Sciences, School of Dentistry, International Medical University, Bukit Jalil 57000, Kuala Lumpur, Malaysia; suanphaik_khoo@imu.edu.my

**Keywords:** TMJ, maxillofacial, cytokines, inflammation, pain

## Abstract

Numerous studies have been conducted in the previous years with an objective to determine the ideal biomarker or set of biomarkers in temporomandibular disorders (TMDs). It was recorded that tumour necrosis factor (TNF), interleukin 8 (IL-8), IL-6, and IL-1 were the most common biomarkers of TMDs. As of recently, although the research on TMDs biomarkers still aims to find more diagnostic agents, no recent study employs the biomarker as a targeting point of pharmacotherapy to suppress the inflammatory responses. This article represents an explicit review on the biomarkers of TMDs that have been discovered so far and provides possible future directions towards further research on these biomarkers. The potential implementation of the interactions of TNF with its receptor 2 (TNFR2) in the inflammatory process has been interpreted, and thus, this review presents a new hypothesis towards suppression of the inflammatory response using TNFR2-agonist. Subsequently, this hypothesis could be explored as a potential pain elimination approach in patients with TMDs.

## 1. Introduction

Temporomandibular disorder (TMD) is a musculoskeletal disorder that is manifested through continuous pain in the temporomandibular joint (TMJ), masticatory muscle, and the periauricular region [1]. Meanwhile, the most common non-odontogenic orofacial pain is a result of the pain in the temporomandibular area [2,3]. Other related symptoms, including hyoid bone tenderness, abnormal swallowing, and tinnitus have an impact on an individual’s sleep, quality of life, and psychological well-being [4]. Therefore, these symptoms also result in depression, stress, anxiety, adverse impacts on energy level, emotional condition and social function [5,6]. The occurrence of TMD symptoms ranges from 21.5% to 50.5%, with a higher occurrence among women compared to men [4]. This difference is due to constitutional, hormonal, psychosocial, biological, anatomical and behavioural factors, although no conclusion has been made [7,8,9]. The etiopathogenesis of TMD has not been specified. Given the perception that the origin of TMD is multifactorial, which includes biopsychosocial, neuromuscular, biomechanical, and biological factors [4,10], treatment for TMD is a multidisciplinary method [11,12,13,14].

TMD does not have a single anatomic origin; generally, it can result from degeneration of the TMJ, painful displacement of the TMJ disc, and pain within the mastication muscles [15]. Degeneration of TMJ may occur via various pathologies, such as osteoarthritis (OA), degenerative joint disease or autoimmune arthritis, as well as mechanical stressors exacerbation [16,17]. Mechanical nociceptor stimulation results in increased rates of neuropeptides, inflammatory mediators and local hypoxia; these changes can lead to pain and dysfunction, potentially exacerbating joint and mastication muscle degeneration and mechanical stress [15,18,19]. Thus, TMD involves joint pain and related myalgia, myofascial pain, tendonitis, spasm, and myositis [20].

**Figure 1:**
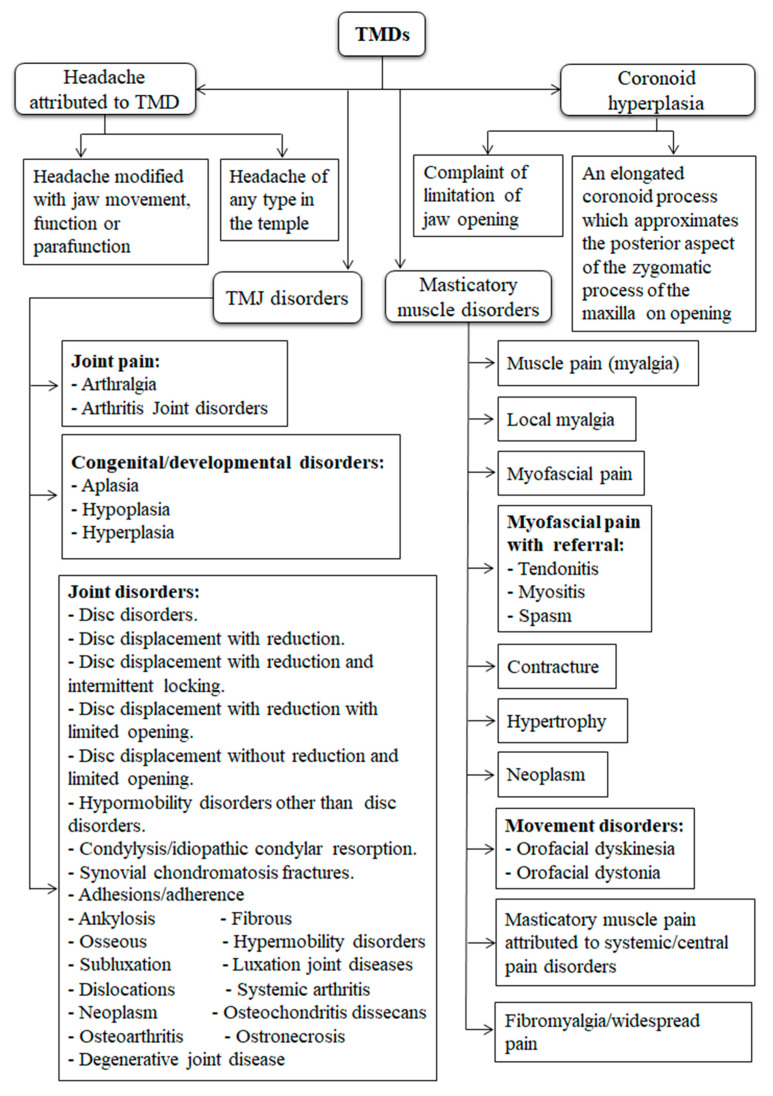
The taxonomic classification of temporomandibular disorders (TMDs). There are 4 main types of TMDs including temporomandibular joint (TMJ) disorders, masticatory muscle disorders, headache attributed to TMD, and coronoid hyperplasia [23,24].

TMD is a heterogeneous category of pathologies as illustrated in Figure 1. Diagnosis of issues related to TMJ is not only challenging, but the proper treatment is also controversial [21]. In addition, the differences in the TMD findings between the individuals at diverse points of time contribute to more challenges in TMD diagnosis [22]. Adequate knowledge is essential to develop a proper treatment in response to the established diagnosis. 

Several issues at the diagnostic level emerged from the heterogeneity of TMD symptoms, which may be indicated through the challenges in treatment planning and responsibility for the taxonomic complexity of such disorders [25]. In most cases, diagnosis is based on history and physical examination. The standard processes come with primary limitations such as the dependency on the human factor (mainly clinicians and patients) [26]. These limitations may lead to misinterpretation of some symptoms and misdiagnosis. Therefore, the development of better approaches is essential to enable the large-scale screening of TMD and specific detection of subjects with or without the disease, which may contribute to development in this area. In addition, the acknowledgement regarding the relationship between TMD and increased degree of biochemical or inflammatory markers enable the exploration of more sensitive and novel diagnostic biomarkers in this field [27]. Accordingly, this article presents a comprehensive review of the recent updates on TMD biomarkers and a promising aspect of the use of biomarkers for the suppression of inflammation and pain elimination, besides case diagnosis.

## 2. TMDs Inflammatory Biomarkers

Biomarker refers to “a characteristic that is objectively measured and evaluated as an indicator of normal biological process, pathogenic processes, or pharmacologic responses to a therapeutic intervention” [28]. Several critical features should be present in an ideal biomarker, such as the required presence in all diagnosed patients (e.g., high sensitivity and specificity), disease specificity, detection before obvious clinical symptoms are present and reversibility after the proper treatment [29]. Furthermore, the ideal biomarkers must not only indicate the seriousness of the illness, but it should also provide information illustrating the cumulative history of the illness and allow a cut-off value with minimal overlap between the normal health condition and disease [30]. In addition, it is predicted that an optimal diagnostic policy, which involves the biomarkers, reduces the overall cost of the diagnosis. In this case, the economic value would comprise the combination of two financially-driven elements, including the costs of measurement and misdiagnosis [31]. 

Following the acquirement of acceptable specificity and sensitivity, a distinguished set of disease biomarkers would contribute to context-relevant, user-friendly and highly simplified methods of TMD diagnosis. However, there is no applicable and direct disease markers panel for TMDs used in the clinical practice as a routine. Adequate progress is present, which indicates the attempts of conducting a thorough evaluation of this field and future research directions based on the previously discussed cumulative evidence. Moreover, several synovial, serum and urinary proteins were found to exhibit important diagnostic tool for TMDs (Table 1). 

Cytokines are the essential polypeptide mediators of critical and severe inflammation [61]. These molecules function as complex immunological networks containing pro-inflammatory cytokines, including interleukin 1 (IL-1), IL-6, and TNF, and anti-inflammatory mediators, such as IL-10 and transforming growth factor-beta (TGF-β) [61]. Despite several controversial results related to cytokines, the high degrees of pro-inflammatory cytokines were generally associated with the TMDs symptoms, including OA and internal derangement (ID) [62]. These mediators contribute to the degradation of cartilage and bone joint by releasing proteinases and other inflammatory molecules. Although pro-inflammatory cytokines are clearly associated with the inflammation of TMJ tissue [62], no explanation has been made regarding the pathophysiology and its mechanism. 

It is noteworthy that IL-8 is another pro-inflammatory cytokine consisting of multifunctional actions in severe and critical inflammation, which indicates the presence of chemotactic activity [62]. Segami and Miyamaru [63] also found a high association between the levels of IL-6 and IL-8 in the synovial fluid of the same patients, indicating that a collaborative activation of theses cytokines is possible in TMDs. Moreover, higher degrees of IL-1α and IL-1β in the synovial fluid of patients with TMD were indicated in various works of research [27]. Meanwhile, few studies examined fibroblasts, which were cultured from TMJ synovial fluid [32,35] and TMJ synovial biopsies (immunohistochemistry) [46,52], leading to consistent results. Furthermore, a study by Ok et al. 2018 [34] demonstrated that the level of urinary pyridinoline (PYD) and deoxypyridinoline (DPD) were increased in patients with TMDs. Additionally, based on the investigation of the blood samples of patients with TMD by Slade and his colleagues, a significant increase was found in the TGF-β1, IL-8, IL-1 receptor antagonist (IL-1ra) and monocyte chemotactic protein (MCP-1) [41]. Overall, it could be seen that these TMD biomarkers have been addressed since 1995; studies have mainly discovered that IL-6, IL-8, IL-1 and TNF were the most potential biomarkers (Table 1). The most important characteristics of these biomarkers are summarized in Table 2. However, due to the disparity among cases and researchers, the results of previous studies could be quite dissimilar even when the same assay methods were used [64], and thus it seems impossible to rely on previous studies in the comparison between the diagnostic and prognostic power of these biomarkers. Further details are provided in the next sections.

### 2.1. IL-8

Among the types of cytokine is IL-8, which is created through macrophages and other cells, including endothelial, airway smooth muscle and epithelial cells [69]. Previously identified as neutrophil-activating protein-1 or monocyte-derived neutrophil chemotactic factor, IL-8 is a chemokine with the ability to induce chemotaxis and active neutrophils [70]. This chemokine was indicated in some types of diseases, especially angiogenic diseases, including rheumatoid arthritis (RA) [71,72]. In this case, IL-8 was found to contribute to the infiltration of neutrophils into the synovial fluid and joint inflammation [71]. It was also illustrated that hypoxia may have a positive impact on the generation of this chemokine [73]. 

Following the inflammation of TMJ with ID, the involvement of IL-8 was assumed [74]. It was also previously found that IL-8 was present at appropriate degrees in the synovial fluid extracted from the patients with ID of the TMJ, including the posterior disk attachment in the patients [74]. Although up-regulation was performed on IL-8 in the inflamed synovial tissues from patients with ID, IL-8 was not indicated in the clinical variables. Furthermore, IL-8 has a secondary function in the pathogenesis of TMJ disorders; the pathological mechanisms leading to the joint disorders are highly complex [75]. Therefore, the possible involvement of IL-8 in the pathological conditions could not be excluded [75]. Further studies were required to specify the potential benefits of IL-8 to the pathological states of patients with ID of the TMJ [75]. Following the inflammation of TMJ in ID cases, IL-8 possibly leads to the induction of inflammatory cells in TMJ [75,76].

### 2.2. IL-6

IL-6 is a circulating cytokine produced by some cells (endothelial cells, adipose tissue, T-cells, smooth muscle cells, and macrophages) [77]. It contributes to myeloid cell differentiation, growth of smooth muscle cells and production of acute-phase proteins [78]. Although IL-6 initiates the reaction of the acute phase and mainly regulates the generation of hepatic C-reactive protein (CRP), there is a constant contradiction regarding IL-6 levels [79]. The association between inflammatory processes and destruction of TMJ elements were illustrated in previous research. As a result, IL-6 was regarded as among the crucial proinflammatory cytokines, which lead to the pathogenesis of TMJ with ID [80,81]. 

With IL-6, the regulation of oncogenesis, hematopoiesis, inflammation, and immunological responses takes place, followed by the mediation of the induction of osteoclast activity and osteoclast progenitor differentiation [81]. Notably, IL-6 is an essential element in the transformation of acute inflammation to chronic, which exhibits a double impact [82]. In this case, this impact may be pro-inflammatory at several degrees and it may also display an anti-inflammatory profile at other degrees [80]. The IL-6 plasma concentrations could be identified within 60 min after the occurrence of tissue injury, with a peak ranging from four to six hours to 10 days [80]. Moreover, IL-6 contributes to the maturation of macrophages, activation, and maturation of neutrophils, and the differentiation/maintenance of cytotoxic T-lymphocytes and natural killer cells [83]. Overall, IL-6 was described in the literature as the major pro-inflammatory cytokines, which results in the pathogenesis of the TMDs and inflammation [27].

### 2.3. IL-1

The IL-1 system consists of a minimum of 21 separate molecules, which lead to the formation of IL-1 receptors, co-receptors, legends, and endogenous antagonists [84]. The IL-1 comprises three categories of legends, namely IL-1α, IL-1β, and IL-1ra. To be specific, IL-1α and IL-1β lead to pro-inflammatory impacts, while IL-1ra prevents the pro-inflammatory functions by playing its role as the competitive receptor inhibitor [85]. Two separate receptors, namely type 1 and type 2 IL-1 receptors, included in IL-1 family [86]. To be specific, type 1 IL-1 receptor plays its role in the induction of intracellular signal transductions after the formation of bond with IL-1, while type 2 IL-1 receptor functions as the decoy receptor [86]. The role of type 2 IL-1 receptor is to form a bond with IL-1 without creating any impacts, which decreases the overall availability of IL-1 for the development and binding of the inflammatory reaction [84]. This type of receptor could also be emitted from the cell surface in a soluble form, which forms a bond with the IL-1 ligands [87]. This bond takes place for the inactivation of the ligands prior to the production of a pro-inflammatory response [88]. Notably, the complex balance between the molecules and receptors of the IL-1 family significantly impacts the TMJ homeostasis, as indicated in many research, which illustrated the presence of higher degrees of IL-1α and IL-1β in the synovial fluid of patients with TMDs [27]. In this study, the synovial fluid samples were extracted from patients with TMDs at diverse phases, which led to the conclusion that the increased degrees of these cytokines result in the development and progress of TMD [89].

### 2.4. TNF

Generally, TNF is regarded as the master pro-inflammatory cytokine [90]. The presence of TNF takes place in the forms of membrane TNF (mTNF), or pro-TNF, and soluble TNF (sTNF) [91,92]. Specifically, mTNF is a transmembrane protein of 26 KDa, which is then transformed into sTNF when divided by TNF-converting enzyme (TACE) [91,93]. Furthermore, TNF plays an essential role in leukocyte recruitment, monocyte chemo-attraction, development of apoptosis, and enhanced management of adhesion molecule expression [94]. Immune cells, such as natural killer (NK) cells, activated macrophages/monocytes and activated T cells, and other non-immune cells, such as fibroblasts and endothelial cells represent TNF [95]. The inflammatory stages lead to the development of TNF as one of the first cytokines, which improves the production of cascade and other inflammatory mediators, such as transcription factors, interleukin IL-1 and IL-6 [96,97]. 

#### 2.4.1. TNF Receptors (TNFRs)

Two categories of TNF receptors (TNFR1 and TNFR2) are restricted at the cellular surface with separate intracellular regions [98]. It was found in a study of the model of inflammation that the up-regulation of TNFR2 was performed over TNFR1, while the treatment with the anti-TNF monoclonal antibody decreased the amount and size of tumour [99]. Therefore, TNF-TNFR2 axis was involved in the suppression of immune response and impacted cell proliferation [100]. Meanwhile, previous studies indicated that the formation of the bond between TNF and TNFR2 was due to stronger connection compared to TNFR1 [101,102]. TNFR1 is the main mediator of TNF-induced apoptosis, which uses its death domain (DD) for the activation of nuclear factor kappa B (NF-κB) pathway [103], while TNFR2 is represented by immunosuppressive cells, especially regulatory T cells (Tregs) [104]. Upon the activation of TNF-TNFR2 axis, the intracellular domains initiate the complexes, which consist of cIAP2, TNF receptor-associated factor-2 (TRAF-2), and cellular inhibitor of apoptosis protein-1 (cIAP-1). As a result, canonical and non-canonical activations of three main pathways take place, which involves mitogen-activated protein kinases (MAPK), activator protein 1 (AP1), and NF-κB pathways [105,106]. These pathways then progress into phosphoinositide 3-kinases/protein Kinase B pathway (PI3K/Akt) signal transduction pathway, which contributes to the development and survival of immunosuppressive cells [107]. 

**Figure 2:**
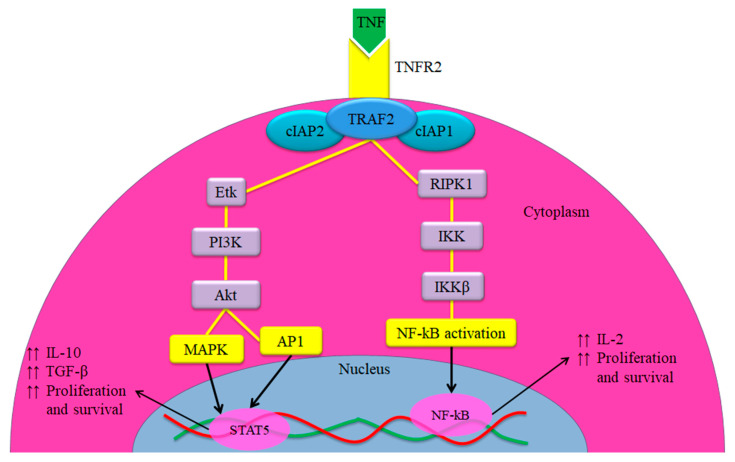
Overview of the TNF-TNFR2 signalling pathway in the immunosuppressive cells. The TNFR2 does not interact with an intracellular DD, while it interacts with complex I that consists of TRAF2 with cIAP1 and cIAP2, and induction of homeostatic signals. The signals travel from complex I either via receptor-interacting serine/threonine-protein kinase 1 (RIPK1) or Etk (a member of the Btk tyrosine kinase family). RIPK1 trigger NF-κB via the IkB kinase (IKK) complex, which results in increasing the transcription of several genes associated positively with cell survival and proliferation. However, the Etk, through the PI3K/Akt pathway, is able to activate both AP1 and MAPK signalling pathways, which activate the promoter of proliferation, survival and other transcription factors. Further, it is associated with enhancing the phosphorylation of signal transducer and activator of transcription 5 (STAT5) that play a crucial role in immunosuppression (Adopted from Al-Hatamleh et al., 2019 [110]).

The NF-κB pathway contributes to the transcription of genes for the spreading and survival of cells [108]. Inflammation is prevented by the suppressive Tregs as the inflammatory cells are suppressed and the tolerogenic dendritic cells (DCs) are induced by the suppressive MDSCs, allowing the suppression of inflammation manifestations [109]. Figure 2 presents a complete description of the implication of TNFR2 activation in building immunosuppressive cells [110].

Essentially, Tregs are prototypical immunosuppressive cells, which reduce the excessive immune reactions and prevent the growth of effector T cell (Teffs) and cytokine to maintain immune homeostasis. In this case, tissue destruction and the growth of autoimmune diseases are inhibited [111]. Tregs could perform the secretion of cytokines, including IL-10, TGF-β and IL-35 or create a direct cell-cell contact to mediate suppressive function [112]. These cells function through the direct suppression of Teffs at the target site and the DC in the regional lymph nodes or direct recruitment of mast cells to the site, which would inhibit the growth of T cells in the regional lymph nodes [113,114]. Therefore, the modulation of TNF-TNFR2 interactions, which are presented on Tregs would be a potential method of enhancing the development of Tregs and suppressing the inflammatory immune reactions.

#### 2.4.2. TNF in TMDs

**Figure 3:**
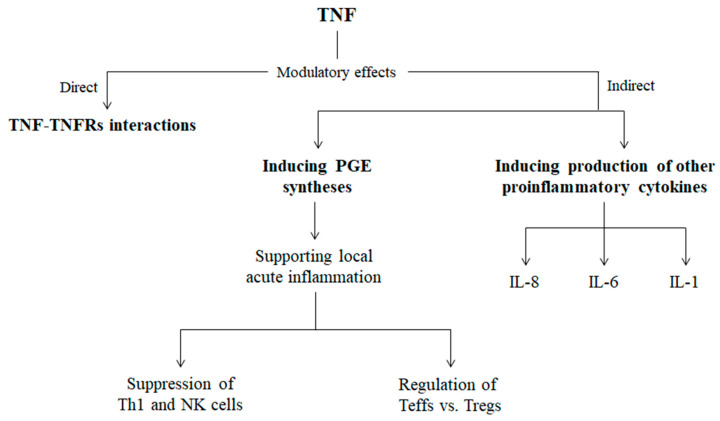
The modulatory effects of TNF on pain and tissue degradation in TMDs. Elevated TNF levels are considered pathological and positively correlated with the inflammatory response and pain in TMDs. The modulatory effects of TNF are divided to direct and indirect effects. The direct effect is based on the interaction of TNF with TNFR1 and TNFR2 as explained in the previous section, while the indirect effect is divided to two main pathways; through inducing production of other proinflammatory cytokines (IL-1, IL-6 and IL-8, mainly) and prostaglandin (PGE) synthesis. PGE in turn plays a vital role in supporting local acute inflammation by regulation of effector T cells (Teffs) vs. regulatory T cells (Tregs), and also by suppression of the T helper 1 (Th1) cells and natural killer (NK) cells [115,117,118].

It was found in a recent study that TMJ pain might have a relation to the deficiency in local cytokine control, which enhanced the inflammatory activity and susceptive to mechanical stimuli over the TMJ [36]. Another study also investigated the influence of TNF in synovial fluid as a determinant of the treatment impact of intra-articular injection of glucocorticoid on TMJ among patients with TMD [115]. This study showed that the high pre-treatment level of TNF was related to the reduction of TNF and absence of pain during the maximal opening of the mouth, while the absence of TMJ pain was related to the reduced TNF levels [115]. A positive relation between TNF levels and TMJ pain was indicated by another research conducted on patients with chronic inflammatory connective tissue disease and its relation to TMJ pain [116]. The proposed mechanisms that correlate the elevated TNF expression with increased TMJ pain are summarized in Figure 3.

#### 2.4.3. TNF-TNFRs Interactions in TMDs

Although the anti-inflammatory soluble TNFR2 (sTNFR2) was within the endogenous cytokine control system and the concentration of it enhanced during inflammation, it was inadequate for the management of inflammation-related increase in TNF levels [119]. Additionally, besides being present in the blood and synovial fluid of the patients with TMJ pain in RA, the sTNFR2 was related to significant joint destruction and inflammatory activity [119]. However, no investigation was conducted on the relative contribution of sTNFR2 and its endogenous control, including articular pain.

An investigation was conducted on severe chronic inflammatory arthritis, which took place in a mice model of RA as a result of the overexpression of TNF [120]. Meanwhile, the enhanced TNFR1 expression was related to the elimination of arthritic impacts, in which the inadequate TNFR2 expression was related to the exacerbated state of arthritic [120,121]. Based on another research conducted on rat TMJ disc cells, which was exposed to cyclic tensile strain, it was found that the expression of TNFR2 was regulated by biomechanical signals regulate. However, this regulation did not occur to TNFR1 under inflammatory conditions [122]. 

New findings were recently developed into the pathogenesis and therapeutic approaches of cartilage degenerative diseases, including TMJ OA [123]. Cortistatin deficiency was associated with OA development. It was found in research on TNFRs-knockout mice that TNFRs was possibly related to the protective role of cortistatin as a cyclic neuropeptide with an endogenous anti-inflammatory potential [123,124]. This research also demonstrated that cortistatin prevented the activation of the NF-κB signalling pathway, which occurred in the TNF-TNFRs interactions and led to the suppression of pro-inflammatory activity on TNF [123,124]. Overall, it was indicated from these limited results that the responses between TNF and TNFR2 among patients with TMDs would be a potential research area for the diagnosis and the management of inflammation and pain.

## 3. Future Directions

Biomarkers appear to be involved in synovial inflammation during internal TMJ derangement, while the association between these levels and TMJ pain level is unclear [125]. Although there are currently no validated pain biomarkers, promising genetic, molecular, neuroradiological, and psychophysical strategies are currently being explored in TMD. Under these conditions, most of the potential biomarkers continue to be in the early stages of discovery and verification. The clinical heterogeneity of pain patient populations, small sample sizes and inadequately characterized TMDs clinical cohorts may present challenges for successful verification and validation of biomarkers. Moreover, it remains to be unclear whether these potential biomarkers can accomplish reliability and practicality considerations (e.g., cost and speed) to bridge the gap from biomarker validation to implementation [126]. 

The synovium facilitates the restriction and concentrations of distinct biomarkers, which presents real-time information regarding joint health [127]. However, the unavailability of the information in relation to blood samples indicates that synovial fluids are possibly optimum for the evaluation of TMDs [128]. Levels of TNF in the TMJ synovial fluid are known to significantly exceed TNF levels in blood plasma samples within the same patient [116]. However, aspiration of the correct volume of synovial fluid from a small joint is a challenging process. Given the importance of the specific equipment and biochemical element, the application of these processes in daily practice is challenging in the practical and technical terms [128]. Although the biomarkers tested from blood may not indicate significant information as those tested from synovial fluids, blood samples must not be disregarded due to it can be used for diagnostic and prognostic in certain circumstance of local and systemic inflammatory disorders.

On the other hand, it is worth to mention that recent in-silico, ex-vivo (human samples) and in-vivo (mouse model) studies have shown TNFR2-agonists as potential approach for the treatment of autoimmune diseases and graft-versus-host disease (GvHD) [129,130,131]. These studies have recorded that TNFR2-agonist activation results in expanding Tregs and thus suppressing the immune response [129,130,131]. Furthermore, there were no clinical trials performed to determine the efficacy of TNFR2-agonists in humans. More clarification is still required to learn how to administer the TNFR2-agonists.

**Figure 4:**
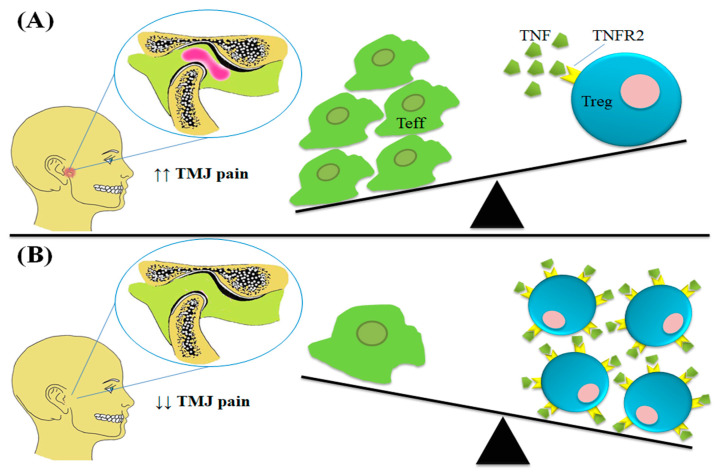
The hypothesized role of TNF-TNFR2 interactions in suppression the inflammatory response in TMDs (Partially adapted from Dimitroulis, 2018 [132]). (**A**) Owning to their role in suppression the immune response (i.e., Teffs), the inhibition of Tregs growth and development expected to be negatively associated with the severity of inflammation and pain in patients with TMD. (**B**) Using of TNFR2-agonists would results in upregulation of TNFR2 on Tregs, and then acceleration of cell growth, development, and extending the immune suppression effects of Tregs. Thus, the weaker inflammatory response will results in pain relief.

We hypothesised that levels of TNFR2 in the patients with TMDs would be constitutively expressed on their Tregs at lower levels. Therefore, the use of potential biological agents (TNFR2-agonist) as immune boosters to maintain the immune homeostasis would be a potential method of enhancing TNFR2 expression. As a result, TNF-TNFR2 interactions would be enhanced leading to further development of Tregs and stronger immune suppression. The TNFR-agonists could also contribute to similar impacts to achieve the main target, immune response suppression, less inflammatory reactions, and pain elimination. The hypothesis is illustrated in Figure 4.

## 4. Conclusions

TMDs are among the most common maxillofacial disorders. Despite the variation of diagnostic procedures, some limitations are still remarkable. The biomarker profile of patients with TMDs, as a critical part of the diagnostic process, is still needed further research to identify gold standard biomarkers. Therefore, this review recommends the TNF and TNFR2 as possible biomarkers not only for diagnostic purposes but also for inflammation and pain relief in patients with TMD. Future extended research is recommended to assess the expression levels of two types of TNFR2 (sTNFR2 and mTNFR2) in TMDs patients’ blood. This review offers a new approach for future studies focusing on targeting the TNF-TNFR2 interactions to suppress the immune response and to reduce the pain in patients with TMDs. 

## Figures and Tables

**Table 1:** List of studies investigated the potential diagnostic markers in patients with TMDs.

Study ID [Reference]	Diagnostic Criteria	Type of Sample(s)	Potential Diagnostic Markers
Xiong et al., 2019 [32]	RDC-TMD and CBCT scan	TMJ synovial fibroblasts	IL-6, leptin and its receptor (Ob-Rb)
Yang et al., 2019 [33]	Wilkes classification	Synovial fluid	IL-8, sTNFR1, sTNFR2 and sIL-6R
Ok et al., 2018 [34]	RDC-TMD	Urine	PYD and DPD
Watanabe et al., 2017 [35]	Arthroscopy of the TMJ	TMJ synovial fibroblasts	IL-1β, IL-6, and IL-8
Ahmed et al., 2015 [36]	The 1987 diagnostic criteria of the American College of Rheumatology.	Synovial fluid and blood	TNF, sTNFR2, and ACPA
Wake et al., 2013 [37]	Clinical symptoms, MRI and arthroscopy	Synovial fluid	Aggrecan, IL-6 and VEGF-A
Nogami et al., 2013 [38]	Panoramic transcranial view and CT scan	Synovial fluid	IL-6
Kim et al., 2012 [39]	RDC-TMD and MRI	Synovial fluid	GM-CSF, INF, IL-1β, IL-2, IL-6, IL-8, IL-10, and TNF-α
Herr et al., 2011 [40]	Wilkes classification	Synovial fluid	EG-VEGF/PK1 and D6
Slade et al., 2011 [41]	RDC-TMD	Blood	MCP-1, IL-1ra, IL-8, and TGFβ1
Kaneyama et al., 2010 [42]	MRI	Synovial fluid	sTNFR1, sTNFR2, sIL-6R, and sIL-1R
Lee et al., 2010 [43]	Clinical symptoms	Synovial fluid	IL-6 and TNF-α
Hamada et al., 2008 [44]	Clinical symptoms and MRI	Synovial fluid	IL-6 and IL-8
Vernal et al., 2008 [45]	Clinical symptoms and MRI	Synovial fluid	IL-1b, IL-2, IL-12p35, IL-12p40, IL-17,IFN-c, TNF-α and TNF-β mRNAs
Kardel et al., 2006 [46]	Clinical symptoms, tomogram	Synovial biopsies	IL-1α, IL-1β and TGF-β
Matsumoto et al., 2006 [47]	And MRI	Synovial fluid	Angiogenin, BDNF, FGF-4, FGF-9, IGFBP-2, IL-8, MIP-1beta, OPG, PARC, TGF-beta2, TIMP-2, and VEGF
Matsumoto et al., 2005 [48]	Clinical symptoms, tomogram, MRI, arthroscopy, lateral oblique and orbit- condylar	Synovial fluid	Angiogenin, FGF-9 and MIP-1β
Kaneyama et al., 2005 [49]	Clinical symptoms and MRI	Synovial fluid	IL-1β, TNF-α, IL-6, sTNFR1, and sTNFR2
Kaneyama et al., 2004 [50]	Clinical symptoms and MRI	Synovial fluid	IL-6 and IL11
Nishimura et al., 2004 [51]	Clinical symptoms and MRI	Synovial fluid	IL-1β and IL-6
Kardel et al., 2003 [52]	Clinical symptoms and tomogram	Synovial biopsies	IL-1α, IL-1β, IFN-γ, and IL-ra
Kaneyama et al., 2003 [53]	Clinical symptoms and MRI	Synovial fluid	OCIF/OPG
Kaneyama et al., 2002 [54]	Clinical symptoms and MRI	Synovial fluid	IL-1β, TNF-α, IL-6, and IL-8
Shinoda et al., 2000 [55]	Clinical symptoms and MRI	Synovial fluid	IL-6 and TIMP-1
Fang et al., 1999 [56]	Clinical symptoms and radiologic examination	Synovial fluid	TGF-β1
Takahashi et al., 1998 [57]	Clinical symptoms, panoramic and transcranial views, tomography, MRI and arthroscopy	Synovial fluid	IL-1β, IFN-γ, and TNF-α
Kubota et al., 1998 [58]	Clinical symptoms and MRI	Synovial fluid	IL-1β and IL-6
Fu et al., 1995 [59]	Clinical symptoms and plain radiograph	Synovial fluid	IL-6
Fu et al., 1995 [60]	Clinical symptoms and plain radiograph	Synovial fluid	TNF-α

PYD: pyridinoline, DPD: deoxypyridinoline, ACPA: anti-citrullinated peptide antibodies, sIL-6R: soluble IL-6 receptor, GM-CSF: granulocyte macrophage colony stimulating factor, EG-VEGF/PK1: endocrine gland-derived vascular endothelial growth factor/prokineticin-1, MCP-1: monocyte chemotactic protein-1, BDNF: brain-derived neurotrophic factor, PARC: pulmonary and activation-regulated protein, OCIF: steoclastogenesis inhibitory factor, OPG: osteoprotegerin, FBG: fibroblast growth factors, TIMP: tissue inhibitors of metalloproteinases, VEGF: vascular endothelial growth factor, RDC-TMD: research diagnostic criteria for TMD, CT: computed tomography, CBCT: cone beam CT, MRI: magnetic resonance imaging. The clinical symptoms include: pain, limitation of mouth opening and clicking.

**Table 2:** A summary comparing the most common biomarkers in TMDs [64,65,66,67,68].

	IL-8	IL-6	IL-1	TNF
Type	Chemokine	Cytokine	Cytokine	Cytokine
Role	Chemoattractant molecule	Signalling molecule	Signalling molecule	Signalling molecule
Sources	T cells, B cells, monocytes, and PMNs	Monocytes and macrophages	Variety of cells, including epithelial cells, macrophages, dendritic cells and B cells	Multiple cell types, including macrophages and T-cells
Signalling pathway(s)	STAT3	MAPK3	NF-κB	NF-κB
Action(s)	Induces chemotaxis and active neutrophils	Induces synthesis of acute phase proteins such as CRP, and inhibits TNF and IL-1 production by macrophages	Initiates and regulates inflammatory responses; IL-1α and IL-1β lead to pro-inflammatory impacts, while IL-1ra prevents it	Regulates immune cells and induce apoptosis

NF-κB: nuclear factor kappa-light-chain-enhancer of activated B cells, STAT3: signal transducer and activator of transcription 3, MAPK3: mitogen-activated protein kinase 3, CRP: C-reactive protein, PMNs: polymorphonuclear leukocytes.
